# Extracranial metastases in glioblastoma—Two case stories

**DOI:** 10.1002/ccr3.1980

**Published:** 2018-12-27

**Authors:** Dorte Schou Nørøxe, Signe Regner Michaelsen, Helle Broholm, Søren Møller, Hans Skovgaard Poulsen, Ulrik Lassen

**Affiliations:** ^1^ Department of Radiation Biology Rigshospitalet Copenhagen Denmark; ^2^ Department of Oncology Rigshospitalet Copenhagen Denmark; ^3^ Department of Pathology Rigshospitalet Copenhagen Denmark

**Keywords:** case stories, extracranial metastases, glioblastoma, gliosarcoma, risk factors

## Abstract

The clinician should always consider extracranial metastases in glioblastoma. Increased risk factors are young age at diagnosis, histology of gliosarcoma, and prior intracranial tumor surgery. Clinical guidelines are needed for this rare event, including consideration for prophylactic intervention.

## 1 INTRODUCTION

Extracranial metastases in glioblastoma (GB) are rare. Here, we present two case stories with metastasis to the spinal cord and the neck musculature, respectively. They both had clinical and histopathological features corresponding to an increased risk of extracranial spread. Clinical guidelines are needed for this rare event.

Glioblastoma, WHO grade IV, is the most aggressive primary brain tumor in adults and has a median overall survival of <15 months despite optimal available treatment.^1^ Although multiple lesion sites can be observed within the brain, extracranial metastases are only rarely seen in 0.4%–0.5% of cases.^2^ Among these, 91% arise from conventional GB, while 9% arise from gliosarcoma (GS),^3^ a variant of GB characterized by having both glial‐ and reticulin‐rich sarcomatous gliomatous components.^4^ The low frequency of extracranial metastases is believed to reflect the short survival as well as intrinsic biological barriers such as dense dura around intracranial veins preventing cell penetration, the blood‐brain barrier (BBB), absence of a genuine lymphatic system in the brain, and lack of a necessary microenvironment for malignant cellular growth in extracranial sites.^2^ Little is known of confounding factors but patients are generally younger^2^, ^5^ and even though there are examples of extracranial metastases at diagnosis before surgery,^6^, ^7^ most patients have had a prior surgical intervention, believed to allow the cancer cells to access the extracerebral blood and lymphatic vessels.^2^, ^5^, ^8^ Meta‐analyses have concluded that the most frequent sites for metastases from GB and GS are lymph nodes, lungs, liver, and bone, but lesions have also been found in skin, spinal cord, and various soft tissue.^3^, ^5^, ^9^ Here, we present two cases of extracranial metastases, one patient with tumor spread/dissemination to the spinal cord from a conventional GB tumor and one presenting subcutaneous spread/infiltration to the neck from a GS, respectively.

## 2 CASE STORY 1

**Figure 1:**
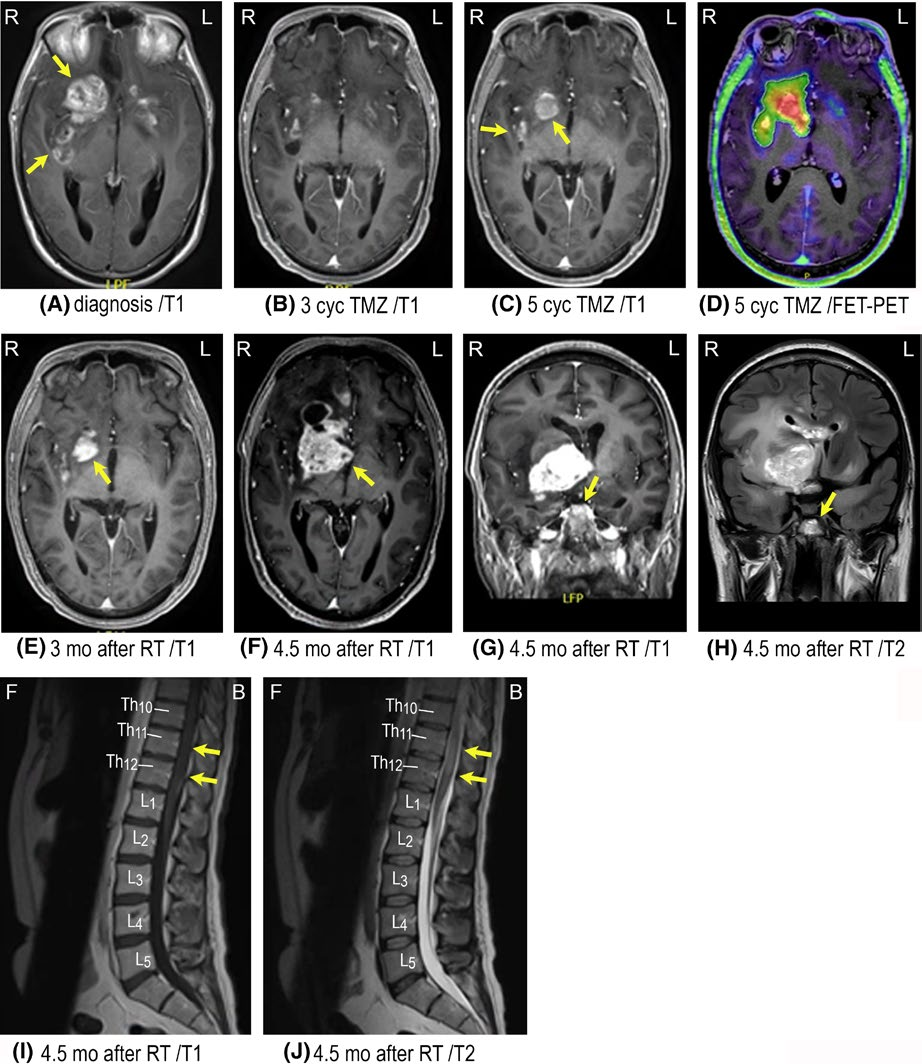
Tumor imaging of Case 1. A, Preoperative T1‐weighted MRI with a 3.3 × 3.6 cm large contrast‐enhanced (CE) tumor involving genu corpus callosum, both frontal lobes, right temporal lobe and both basal nuclei including subependymal lesions in the left frontal horn. B, T1‐weighted MRI after three series of temozolomide (TMZ), showing regression. C, T1‐weighted MRI after five series of TMZ with suspicion of progressive disease (PD). D, FET/PET with confirmation of PD with increased metabolic activity in the tumor. E, T1‐weighted MRI of tumor regression after radiotherapy. F‐H, PD in all lesions including spread to the fourth ventricle shown on both T1 and T2‐FLAIR MRI. I‐J, Non‐CE tumor in the spinal cord at levels TH11/Th12 on T1‐ and T2‐weighted MRI. Body orientation is indicated by R (right), L (left), F (front), and B (back)

A 30‐year‐old male was brought to the department of neurosurgery due to a history of confusion, headaches, nausea, seizures, and insomnia which had accelerated during the last 30 days. The patient had a history of systemic infection with Epstein‐Barr virus (EBV) 5 years previously which had been treated conservatively. At admission, a Glasgow Coma Scale (GCS) of 11 was noted (eye: 2, verbal: 3, motor: 6). A magnetic resonance imaging (MRI) of the brain (Figure 1A) showed a 3.3 × 3.6 cm large tumor involving genu corpus callosum, both frontal lobes, right temporal lobe and both basal nuclei including subependymal lesions in the left frontal horn. Hence, a large multifocal, infiltrating tumor was found with surrounding edema. High‐dose corticosteroids and anticonvulsants were started, including thiamine and B‐vitamins due to suspicion of alcohol abuse. No computed tomography (CT) of the body was done but in relation to the EBV‐infection 5 years prior to diagnosis, a CT of the thorax and abdomen had shown no suspicion of malignancy. A stereotactic biopsy was done, and the histopathological examination confirmed the diagnosis of GB with mitosis, microvascular proliferation, necrosis, and positivity for the astrocytic marker glial fibrillary acidic protein (GFAP; Figure 3A,B). Immunohistochemistry analysis found isocitrate dehydrogenase (IDH) and alpha‐thalassemia mental retardation syndrome (ATRX) mutation, suggesting a secondary GB. By polymerase chain reaction (PCR), the tumor was found O‐6 methyl‐guanine‐DNA‐methyl‐transferase (MGMT) promotor methylated. No 1p/19q codeletion was found. Due to the extent of the tumor, a gross resection was not possible without serious impairment of vital functions and the patient was referred directly to the oncology department for further treatment. Upfront concurrent radiotherapy also was not possible since the size of the radiation field would be too extensive. Hence, the patient was started on temozolomide (TMZ) monotherapy with the hope of minimizing the tumor for later radiation. After three cycles of TMZ, MRI (Figure 1B) showed almost complete remission of all measurable lesions and the patient continued TMZ. Following two more cycles of TMZ, a new MRI (Figure 1C) showed progression of the contrast‐enhanced tumor components in the right basal frontal lobe, basal nucleus area, insula, and a new lesion in the left basal nucleus area. A positron emission tomography (PET) with the radiolabeled O‐(2‐18F‐fluoroethyl)‐L‐tyrosine (FET) (Figure 1D) found metabolic activity in the described areas, hence a very progressive relapse. A tumor board review advised against relapse surgery and the patient started on radiation therapy with 60 Gy/30F, 5F/W. Three months after radiation, a follow‐up MRI showed regression of the contrast‐enhanced lesions and no new lesions (Figure 1E). After 1½ months, the symptoms recurred, and the patient was hospitalized. MRI (Figure 1F‐H) showed massive progression in all areas, including the fourth ventricle and the patient developed symptoms of cauda equina syndrome with paresis of both legs, lack of deep reflexes, decreased muscle tone, insensibility and loss of pinch function. An MRI of the spine (Figure 1I,J) confirmed the clinical diagnosis with hyperintensity at levels Th11/Th12 and contrast enhancement at several levels in the spinal cord. High‐dose corticosteroids and radiation therapy at Th11/Th12 with 25 Gy/5F, 5F/W were prescribed. The patient completed the treatment but deteriorated and died 1 week later, 12.5 months after diagnosis.

## 3 CASE STORY 2

**Figure 2:**
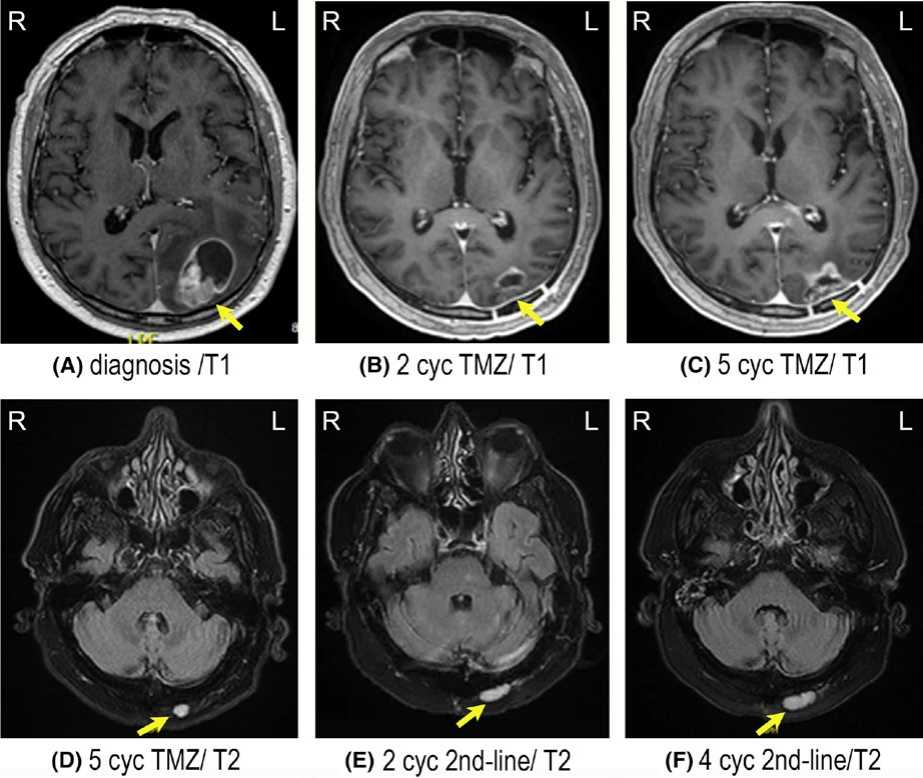
Tumor imaging of Case 2. A, Preoperative T1‐weighted MRI showing a cystic, multi‐lobular, contrast‐enhanced 2.5 × 4.7 cm large lesion in the left parietal lobe. B, Stable disease (SD) after two series of adjuvant temozolomide (TMZ) on T1‐weighted MRI. C and D, Progressive disease (PD) after five cycles of adjuvant TMZ with a new lesion in the neck on T1 and T2‐FLAIR MRI. E, T2‐FLAIR MRI after two cycles of bevacizumab (BEV)/CPT11, SD at the primary tumor site but PD in the tumor in the neck. F, T2‐FLAIR MRI after four cycles of BEV/CPT‐11 finding SD at the primary tumor site but PD of the tumor in the neck. Body orientation is indicated by R (right) and L (left)

**Figure 3:**
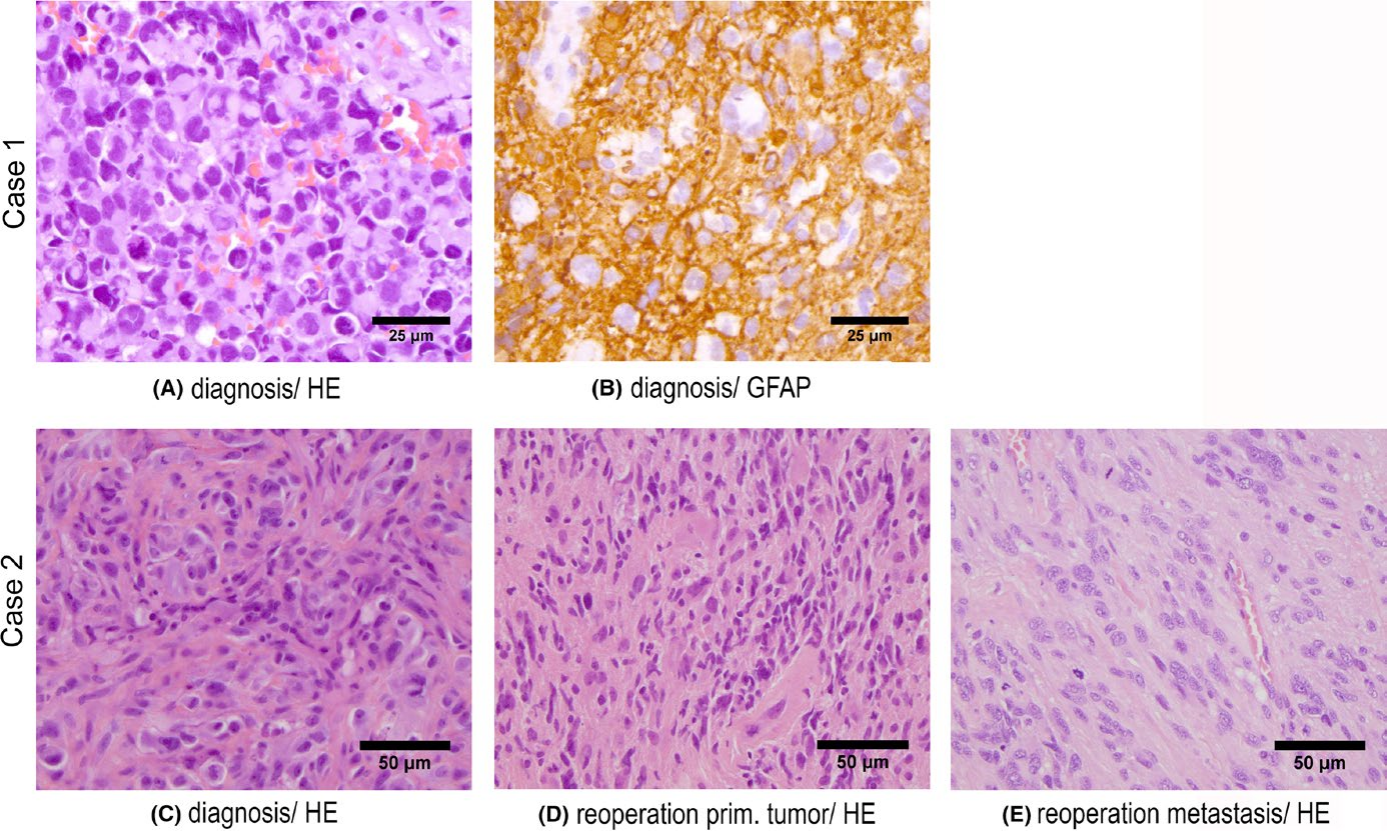
Immunohistochemical images of resected tumors. A, Case 1, Hematoxylin and eosin (HE) staining of the diagnostic tumor material. B, Case 1, glial fibrillary acidic protein immunoreactivity in the diagnostic tumor material. C, Case 2, HE of diagnostic tumor material. D, Case 2, HE of tumor from reoperation from the primary site in the brain. E, Case 2, HE of metastasis located in patient's neck

A 62‐year‐old male with a former history of arthritis and age‐related macular degeneration was admitted to the emergency room with confusion and acute expressive and partly impressive aphasia. The patient had experienced a similar attack 1 month earlier which had resolved spontaneously. An MRI of the brain (Figure 2A) found a cystic, multi‐lobular, contrast‐enhanced 2.5 × 4.7 cm large lesion in the left parietal lobe. High‐dose corticosteroid was started, and the patient had a gross resection of the tumor. Histopathological examination revealed mitosis, microvascular proliferation, necrosis, and sarcomatous tumor growth (Figure 3C). Immunohistochemistry found IDH‐wildtype (WT) and ATRX‐WT. PCR showed a nonmethylated MGMT promotor, consistent with GB GS. Concurrent chemo/radiation with TMZ was started. The patient completed the treatment without complications and MRI (Figure 2B) showed stable disease after two cycles of adjuvant TMZ. After five cycles of adjuvant TMZ, the patient complained of nausea and dizziness and the MRI (Figure 2C,D) showed progression corresponding to the surgical cavity and a new lesion was found in the left neck region. This was confirmed by a FET/PET (not shown) with increased metabolic activity in the same areas. A subtotal resection, including the tumor in the neck, was done. Not all the tumor in the neck could be removed due to infiltrative growth in the musculature. Histopathology of tissue from both the brain and neck confirmed relapse of the formerly diagnosed GB (Figure 3D,E). Second‐line therapy was started with bevacizumab (BEV) and irinotecan (CPT11). Due to a new attack with aphasia and seizure in the right hand, the patient was started on anticonvulsants. The clinical condition deteriorated, and the oncologic treatment was stopped in accordance with the patient's wishes. MRI (Figure 2E,F) during the BEV/CPT11 treatment showed stable disease in the brain (not shown) but a progression of the lesion in the neck. The patient was no longer candidate for systemic treatment and he declined palliative irradiation against the area in the neck. The patient died 15 months after the diagnosis.

## 4 DISCUSSION

Both patients presented in this study had a survival after diagnosis consistent with the literature.^1^ Hence, the extracranial metastases did not significantly shorten their lives.

The patient in case story 1 had upfront risk factors for extracranial metastases with young age at diagnosis and surgery (biopsy).^2^, ^5^ Moreover, this patient had a multifocal, bihemispheric tumor that precluded concurrent chemoradiation. On the other hand, the tumor was MGMT methylated, found to be predictive for better effect of TMZ,^10^ as also observed at start of treatment in this case. We hypothesize that the tumor had been present for several years before diagnosis and hence had time to develop subclones suitable for metastasizing. We base this on the year‐long history with lack of initiative, forgetfulness, and inability to maintain a stable job nor a place to live since the age of 25. These deficiencies worsened when he—at the age of 30—was diagnosed with what presumably was a secondary GB, based on the presence of both IDH and ATRX mutation.^11^

The primary tumor involved the right frontal horn in the lateral ventricle and had spread to the fourth ventricle at progression. The ventricles communicate to the spinal cord and this could represent the metastatic route. Metastases to the bone are found in approximately 31% of all extracranial metastases but when it occurs, the most common site is the vertebrae (73% of cases).^12^ Whether these vertebral metastases described in the literature have involved the ventricles of the brain is unknown, but this might be the case due to the direct contact between tumor cells and the cerebrospinal fluid running in the subarachnoid space in the spinal cord. Circulating tumor cells present in the ventricular system could potentially seed in the vertebrae, as supported by observations of metastases to the abdomen in patients with a ventriculo‐peritoneal shunt.^13^

The role of IDH has been known since 2008 and was implemented at our institution in 2009. But IDH status was not integrated into the World Health Organization (WHO) classification before 2016^11^ and the role of IDH in extracranial metastases can be unclear because of few cases. Since IDH‐mutation is correlated with young age at diagnosis, there is a presumption that a GB with extracranial metastases could have an overrepresentation of IDH mutations, but this is unknown.

For the patient in case story 2, the tumor in the neck was not seen until after surgery, confirming the increased risk of extracranial metastases after surgery with iatrogenic seeding. The patient had a GS described to be associated with increased risk for development of extracranial metastases.^9^, ^14^ Therefore, in the case of GSs, expansion of the radiation field around the tumor and also to the cranial entrance of surgery could possibly reduce the risk of infiltrative metastases outside the cranial cavity.

When the tumor in the neck was diagnosed with the same histology as the primary tumor, the patient was offered palliative radiotherapy against the neck since he was not a candidate for systemic treatment, but he declined. This underlines the discussion for which palliative strategies to turn to since patients at the time of diagnosis of extracranial metastases in general have limited lifespan left of a few months.^2^ A discussion with the patient and the family should highlight the needs and wishes for how to spend the rest of the life and focus should always be on quality of life instead of active treatment strategies at this stage.

Although rare, the incidence of extracranial metastasis from malignant gliomas is increasing,^3^ presumably related to the prolongation of patient life with better treatment options combined with better imaging during treatment which diagnoses the asymptomatic extracranial lesions. Still, the literature has documented <200 case stories of extracranial metastases in GB/GS,^3^ but the real number might be even higher since these patients may not experience symptoms from the peripheral metastasis but die from the progression of the intracranial lesion. We speculate that more asymptomatic metastases would be found if more patients with GB would undergo CT of the thorax and abdomen and postmortem autopsy. To our knowledge, there are no clinical guidelines for extracranial metastases in GB. We suggest that a histological examination of the extracranial metastases is always conducted including at postmortem autopsies, being in line with the diagnostic criteria for extracranial metastases proposed by Weiss et al^15^ in 1955. With the easier access to whole exome/genome sequencing and expression analyses, making a genomic profile of both the primary and the metastatic tumor whenever possible could also be beneficial in order to identify genes of interest to be involved in the metastatic process. Furthermore, it would be interesting to set up a research project, investigating for circulating tumor cells in the cerebrospinal fluid with a lumbar puncture if the patient has a tumor infiltration to the ventricles. This might help identify patients eligible for prophylactic spinal irradiation. These above suggestions could contribute to the development of a meaningful clinical guideline for extracranial metastases.

## 5 CONCLUSION

The presented cases in this study support young age at diagnosis, prior intracranial brain tumor surgery, and GS components in the tumor as risk factors for the development of extracranial metastases. We suggest that more extensive radiation fields should be considered in the case of GS tumors to minimize extracranial spread. Moreover, we find a need for the development of clinical guidelines to detect extracranial spread in malignant glioma to improve treatment and to better understand this phenomenon.

## CONFLICT OF INTEREST

None declared.

## AUTHOR CONTRIBUTIONS

DSN, HSP, and UL: Concepted the study. DSN and SRM: Evaluated the case materials and wrote the manuscript. HB: Contributed to the histological evaluation. SM: Contributed to the imaging evaluation. All authors participated in critical review and revision of the manuscript and gave approval of the final manuscript.

## APPROVAL

The project has been approved by the Danish Data Protection Agency with the journal number: VD‐2018‐459.
